# Retrospective Cohort Study of Lassa Fever in Pregnancy, Southern Nigeria

**DOI:** 10.3201/eid2508.181299

**Published:** 2019-08

**Authors:** Sylvanus Okogbenin, Joseph Okoeguale, George Akpede, Andres Colubri, Kayla G. Barnes, Samar Mehta, Reuben Eifediyi, Felix Okogbo, Joseph Eigbefoh, Mojeed Momoh, Mojeed Rafiu, Donatus Adomeh, Ikponmwosa Odia, Chris Aire, Rebecca Atafo, Martha Okonofua, Meike Pahlman, Beate Becker-Ziaja, Danny Asogun, Peter Okokhere, Christian Happi, Stephan Günther, Pardis C. Sabeti, Ephraim Ogbaini-Emovon

**Affiliations:** Institute of Lassa Fever Research and Control, Irrua Specialist Teaching Hospital, Irrua, Nigeria (S.A. Okogbenin, J. Okoeguale, G. Akpede, R. Eifediyi, F. Okogbo, J. Eigbefoh, M. Momoh, M. Rafiu, D. Adomeh, I. Odia, C. Aire, R. Atafo, M. Okonofua, D. Asogun, P. Okokhere, E. Ogbaini-Emovon);; Ambrose Alli University Faculty of Clinical Science, Ekpoma, Nigeria (S.A. Okogbenin, G. Akpede, D. Asogun, P. Okokhere),; Broad Institute of MIT and Harvard, Cambridge, Massachusetts, USA (A. Colubri, K.G. Barnes, S. Mehta, P.C. Sabeti);; Harvard University, Cambridge (A. Colubri, P.C. Sabeti);; Beth Israel Deaconess Medical Center, Boston, Massachusetts (S. Mehta);; Bernhard Nocht Institute for Tropical Medicine and German Centre for Infection Research, Partner Site, Hamburg, Germany (M. Pahlman, B. Becker-Ziaja, S. Günther);; Department of Biological Sciences and African Center of Excellence for Genomics of Infectious Diseases, Redeemer’s University, Ede, Nigeria (C. Happi);; Harvard School of Public Health, Boston (P.C. Sabeti)

**Keywords:** Lassa fever, pregnancy, southern Nigeria, West Africa, vector-borne infections, hemorrhagic fever, viruses

## Abstract

Lassa fever in pregnancy causes high rates of maternal and fetal death, but limited data are available to guide clinicians. We retrospectively studied 30 pregnant Lassa fever patients treated with early ribavirin therapy and a conservative obstetric approach at a teaching hospital in southern Nigeria during January 2009–March 2018. Eleven (36.7%) of 30 women died, and 20/31 (64.5%) pregnancies ended in fetal or perinatal loss. On initial evaluation, 17/30 (56.6%) women had a dead fetus; 10/17 (58.8%) of these patients died, compared with 1/13 (7.7%) of women with a live fetus. Extravaginal bleeding, convulsions, and oliguria each were independently associated with maternal and fetal or perinatal death, whereas seeking care in the third trimester was not. For women with a live fetus at initial evaluation, the positive outcomes observed contrast with previous reports, and they support a conservative approach to obstetric management of Lassa fever in pregnancy in Nigeria.

## Abstract 2

The positive outcomes observed support a conservative approach to disease management.

Lassa fever (LF), a viral hemorrhagic fever endemic to West Africa (*1*–*3*), was first reported in 1969 from northern Nigeria (*4*,*5*). Since that time, LF has been documented in several countries in West Africa, including Sierra Leone, Liberia, Guinea, Mali, and, more recently, Benin and Togo (*6*–*9*). Historical reports of LF in pregnancy have described poor maternal and fetal outcomes; an early direct comparison in Sierra Leone reported a 50% mortality rate in pregnant women (n = 30), compared with 16% among nonpregnant women (n = 234) (*10*). A subsequent study conducted in Sierra Leone in 1988 observed a smaller disparity of a 21% mortality rate in pregnant women (n = 68) versus 13% among nonpregnant women (n = 79) but found a worse mortality rate (30%, n = 40) for mothers in the third trimester and an overall fetal and neonatal mortality rate of 87% (*11*). Although the Sierra Leone study demonstrated the considerable contribution of LF to overall maternal mortality rates at a single hospital, since then, large studies of pregnant patients with LF have been lacking, leading to difficulty in estimating the actual regional burden on maternal health. This problem is exacerbated by the large variability in Lassa virus across regions (*12*) and the often nonspecific early signs and symptoms of the disease, including overlap with other common infectious diseases in the region, such as malaria, influenza, and bacterial sepsis (*2*,*13*).

Pathophysiologically, the poor outcome of LF in pregnancy has been attributed to the higher viral loads often observed in pregnant compared with nonpregnant patients, possibly because of the poorly understood immunologic changes in pregnancy or the affinity of the virus for the highly vascularized placenta (*14*,*15*). Also, the overlap of symptoms such as nausea, headache, and abdominal pain with complicated or even uncomplicated pregnancy might further delay identification or diagnosis and result in worse outcomes when the infection is severe (*16*).

The management of LF in pregnancy requires making difficult decisions with sparse data for guidance. The antiviral drug ribavirin represents the only established pharmacologic therapy for LF and is believed to substantially reduce overall mortality rates (*17*,*18*), although the mechanism of action is not clear and data on safety of the drug in pregnancy are limited (*19*). The 1988 Sierra Leone study, which has been the largest LF case series in the literature to date, found that delivery, spontaneous abortion (miscarriage), and evacuation of the uterus all improve maternal outcome (*11*); the study recommended active obstetric management, particularly because the authors observed high fetal mortality rate irrespective of the modality of management (*2*). However, patients in the study did not receive ribavirin before delivery because of published evidence of teratogenicity in animal studies (*19*). To improve maternal and fetal outcomes and explore their relationship to clinical signs and symptoms, we retrospectively analyzed >9 years of records at a hospital in Nigeria that treated a substantial number of LF cases and applied both a more conservative approach to obstetric management and more liberal antepartum use of ribavirin.

## Methods

Irrua Specialist Teaching Hospital (ISTH) is a tertiary-care federal hospital that served as the LF national referral center for the duration of our study. Providers at ISTH and outside facilities in >30 states refer specimens for Lassa virus (LASV) testing by reverse-transcription PCR (RT-PCR) for patients who fit our previously described case definition (*20*); outside patients who test positive by RT-PCR are often referred to ISTH for management. Two authors (S.O. and J.O.) reviewed and abstracted from hospital medical records clinical data for every laboratory-confirmed case of LF in a pregnant woman eventually admitted to ISTH during January 2009–March 2018. During this period, 44 pregnant women were managed for LF. On initial review of these patient records, we found 30 patients for whom documentation of signs and symptoms at admission and during hospitalization were sufficiently complete for inclusion in this study. Comparison data for facility maternal mortality rates over the same period was based on summaries maintained by the ISTH Department of Obstetrics and Gynaecology.

During the study period, pregnant women with confirmed LF received the same supportive care as nonpregnant LF patients, including intravenous fluids (either normal saline or lactated Ringer solution) to improve organ perfusion. All patients had antimicrobial prophylaxis with broad-spectrum antibiotics (ceftriaxone in most cases) and antimalarial drugs (typically oral artemether/lumefantrine; when required, parenteral treatment was quinine before 2014 and artemether after 2014). Fifteen of the 30 pregnant women in our series received blood transfusions for anemia (defined as packed red cell volume <25%), and 2 women required renal dialysis (they were referred for nephrologic evaluation when urine output was <0.5 mL/kg/h, although final decision to dialyze was clinical).

All patients were treated with intravenous ribavirin, except 2 patients who died shortly after arrival. Intravenous ribavirin treatment was started with a 100 mg/kg loading dose (two thirds dose on admission, one third 8 hours later), then 16 mg/kg every 6 hours for 4 days, followed by 8 mg/kg every 8 hours for 6 days. All pregnant women had an ultrasound scan on arrival to confirm fetal cardiac activity. After admission, fetal well-being was monitored at least 2 times each week by using either Doppler velocimetry, a nonstress test, or a biophysical profile, as appropriate for gestational age. We used a conservative obstetric management strategy throughout, only inducing labor in cases of fetal compromise and only performing uterine evacuation in women with fetal death. When uterine evacuation was required, we initiated vaginal misoprostol, dosed as appropriate for gestational age and consistent with International Federation of Gynecology and Obstetrics guidelines (*21*,*22*), followed by manual vacuum aspiration and curettage when required.

Before 2014, women who cleared viremia on the basis of RT-PCR results were discharged home and had outpatient weekly follow-up, including a nonstress test or biophysical profile. After 2014, in response to a case of late intrauterine fetal death after discharge, women were kept as inpatients for close monitoring until delivery. In all cases, patients had postpartum follow-up at 2 and 6 weeks after delivery. Newborns were not discharged until >1 week after delivery, in accordance with hospital protocol for LASV-exposed infants.

For our study, we defined the first trimester as <14 weeks from the first day of the last menstrual period (LMP), the second trimester as 14 weeks to <28 weeks from LMP, and the third trimester as >28 weeks from LMP. We defined stillbirth as delivery after 28 weeks’ gestation of a baby without signs of life (i.e., absent cardiac and respiratory activity). Perinatal mortality rate attributable to LF was defined as the proportion of all pregnancies with laboratory-confirmed maternal diagnosis of LF that ended in either intrauterine fetal death after 28 weeks, stillbirth, or neonatal death in the first week of life. Overall maternal mortality rate was defined as the proportion of women who died while pregnant or within 42 days of the end of pregnancy, irrespective of the duration or site of the pregnancy, from causes related to or aggravated by pregnancy or its management, but not from accidental or incidental causes (*23*). When discussing maternal mortality rates related to LF in the context of this study, we refer to the proportion of women in whom LF was diagnosed during pregnancy and who did not survive that pregnancy.

For statistical analysis, we calculated the mean + SD for quantitative variables, whereas we used prevalence to characterize qualitative variables. We calculated the univariate correlation between clinical features (signs and symptoms) with maternal and fetal death and then ranked these features by the p value of their association with maternal outcome (either death or survival) by using a χ^2^ test with Yates correction. Tests of statistical significance (defined as p<0.05) were based on a 95% CI.

## Results

Forty-four cases of LF in pregnancy were managed at ISTH during the study period, out of a total of 5,048 pregnant women admitted to ISTH during that period. The 30 women for whom we were able to recover complete records were 16–39 years of age (mean + SD 28.1 + 5.1 years). These 30 women were admitted at gestational ages of 5–39 weeks (mean 21.6 + 10.6 weeks) (Table 1). Parity ranged from 0–6 (mean 3.0 + 1.6). One woman had a twin pregnancy.

**Table 1:** Demographic characteristics and outcomes observed in a retrospective cohort study of Lassa fever in pregnancy conducted at Irrua Specialist Teaching Hospital, Irrua, Edo State, Nigeria, January 2009–March 2018*

Year of diagnosis	Maternal age, y	Fetal gestational age, wks	Maternal clinical status†	Fetal status	Maternal outcome	Perinatal outcome
2009	24	10	Severe	Dead	Died	NA
2009	35	6	Severe	Dead	Died	NA
2009	28	12	Severe	Dead	Survived	NA
2009	29	7	Severe	Dead	Survived	NA
2009	31	22	Severe	Dead	Died	NA
2010	16	33	Mild†	Alive	Survived	Survived
2010	26	32	Severe	Dead	Died	NA
2010	37	11	Severe	Dead	Died	NA
2010	30	31	Severe	Dead	Survived	NA
2011	26	24	Severe	Dead	Died	NA
2011	26	9	Severe	Dead	Died	NA
2011	32	36	Mild	Alive	Survived	Survived
2011	27	11	Severe	Dead	Survived	NA
2012	39	35	Mild	Alive	Survived	Died 3 d after birth
2012	26	33	Mild	Alive	Survived	Survived
2013	25	8	Severe	Dead	Died	NA
2013	30	31	Mild	Alive	Survived	Survived
2013	31	29	Mild	Alive	Died	Died at 38 wks
2013	32	10	Severe	Dead	Survived	NA
2014	18	33	Mild	Dead	Survived	NA
2014	28	12	Severe	Dead	Survived	NA
2015	26	28	Mild	Alive	Survived	Survived
2016	33	27	Mild	Alive	Survived	Survived
2017	35	34	Mild	Alive	Survived	Survived
2017	32	29	Mild	Alive	Survived	Twins; 1 died at 37 wks, 1 survived
2017	23	32	Severe	Dead	Died	NA
2017	24	31	Mild	Alive	Survived	Survived
2017	36	25	Severe	Dead	Died	NA
2018	33	29	Mild	Alive	Survived	Survived
2018	32	33	Severe	Alive	Survived	Survived

*NA, not applicable. †Severe maternal outcome defined as any of the following: convulsions, irrational behavior, coma, extravaginal bleeding, or oliguria (<0.5 mL/kg/h for ≥6 h). Mild maternal presentation defined as the absence of all of these features.

We divided clinical signs and symptoms into 2 broad patterns. First, 16 women had complications, defined as either coma, convulsions, irrational behavior, extravaginal bleeding, or oliguria. All of these 16 women were also found to have intrauterine fetal death or an abortive process. Maternal death ensued within 24–48 hours in 10/16 (58.8%) of these cases and, in most cases, uterine evacuation was not completed before death. The second clinical pattern was observed in 14 women who had with milder, nonspecific symptoms, including fever, malaise, cough, and sore throat. Thirteen of these 14 women had a live fetus on ultrasound, and only 1 (7.1%) of the 14 women died. Irrespective of initial signs and symptoms, in all cases of intrauterine fetal death, uterine evacuation was initiated using misoprostol vaginal tablets as we described. Four of these patients died before complete evacuation, and 5 women required manual vacuum aspiration for evacuation of retained products of conception.

All 30 women had fever. Overall, 19 women survived and 11 died, resulting in an overall LF-related maternal mortality rate of 36.7% in this cohort. Seven of the 11 deaths occurred within 24 hours of admission, and 4 occurred within 24–48 hours of admission. Fetal death in utero, retrosternal chest pain, vomiting, and cough were the most frequent clinical features apart from fever, whereas deafness was the least frequent (Table 2). Extravaginal bleeding, convulsions, oliguria, fetal death in utero, and cough were each significantly associated with maternal death. In contrast, admission with a live fetus and breast pain or engorgement were each significantly associated with survival.

**Table 2:** Relationship between clinical manifestations and maternal death observed in a retrospective cohort study of Lassa fever in pregnancy conducted at Irrua Specialist Teaching Hospital, Irrua, Edo State, Nigeria, January 2009–March 2018*

Sign or symptom	No. (%)			p value	OR (95% CI)
	All women, N = 30	Women who survived, n = 19	Women who died, n = 11		
Retrosternal pain	19 (63)	13 (68)	6 (54)	0.7	0.55 (0.12–2.6)
Vomiting	18 (60)	12 (63)	6 (54)	0.7	0.7 (0.15–3.2)
Headache	16 (53)	13 (68)	3 (27)	0.06	0.17 (0.03–0.89)
**Fetal death**	17 (56)	7 (36)	10 (90)	**0.007**	**17 (1.8–>100)**
Vaginal bleeding	14 (46)	6 (31)	8 (72)	0.06	5.8 (1.1–30)
**Breast pain or engorgement**	13 (43)	12 (63)	1 (9)	**0.007**	**0.06 (0.01–0.56)**
Difficulty swallowing	12 (40)	6 (31)	6 (54)	0.3	2.6 (0.56–12)
Sore throat	11 (36)	6 (31)	5 (45)	0.7	1.8 (0.39–8.3)
Abdominal pain	10 (33)	6 (31)	4 (36)	1	1.2 (0.26–5.9)
**Cough**	10 (33)	3 (15)	7 (63)	**0.01**	**9.3 (1.6–53)**
**Extravaginal bleeding**	9 (30)	0 (0)	9 (81)	**<0.0001**	**>100 (0–>100)**
Renal angle tenderness	9 (30)	6 (31)	3 (27)	1	0.81 (0.16–4.2)
**Convulsions**	8 (26)	1 (5)	7 (63)	**0.001**	**31 (3–>100)**
**Oliguria**†	8 (26)	1 (5)	7 (63)	**0.001**	**32 (3–>100)**
Preterm contractions	8 (26)	7 (36)	1 (9)	0.2	0.17 (0.02–1.6)
Jaundice	7 (23)	2 (10)	5 (45)	0.07	7.1 (1.1–47)
Deafness	5 (16)	3 (15)	2 (18)	1	1.2 (0.17–8.5)

*Boldface indicates a statistically significant correlation between a sign or symptom and death (p<0.05 by χ^2^ or Fisher exact test for maternal death among women with a stated sign or symptom compared with women without that sign or symptom). OR, odds ratio. †Defined as <0.5 mL/kg/h for ≥6 h.

The mean + SD duration of symptoms before admission was 6 + 3.1 days among the survivors and 10 + 3.5 days among those who died. When divided by trimester of pregnancy, maternal mortality rate was 50.0% (5/10) in the first, 75.0% (3/4) in the second, and 18.7% (3/16) in the third trimester. We noted a trend toward a lower maternal mortality rate in the third trimester, but this difference did not reach statistical significance (p = 0.06). Overall, deaths attributed to LF accounted for 13.1% (11/84, out of 5,048 total admissions of pregnant women) of maternal deaths at ISTH during the study period, second only to postpartum hemorrhage (28.5%) and eclampsia (22.6%).

Overall, 17/31 (56.7%) of fetuses had died by the time of admission. Of the 13 women with viable pregnancies at admission, 1 had a twin gestation and subsequently delivered 1 live baby (Apgar score 4 at 1 minute, 8 by 5 minutes) without apparent abnormality and 1 stillbirth at term. Another patient returned with delayed intrauterine fetal death at 38 weeks’ gestation, despite having cleared her viremia after initial examination 9 weeks earlier, and died shortly after the return admission; a repeat LASV RT-PCR test was not performed. Five of the women with viable pregnancies at discharge had full-term deliveries, 5 had preterm deliveries at 33–36 weeks’ gestation, and 1 had early neonatal death on the third day of life. The rate of perinatal death or spontaneous abortion in women who were admitted with a live fetus was 21.4% (3/14), compared with an overall rate of fetal and perinatal loss of 64.5% (20/31). In relation to the fetus, the mortality rate was significantly lower when the mother was admitted to the hospital in the third trimester; the rate was 31.2% (6/17) for the third trimester compared with 92.9% (13/14) for the first and second trimesters combined (p<0.001). Maternal signs and symptoms that included extravaginal bleeding, convulsions, oliguria, or vaginal bleeding were each associated with fetal death (p<0.01) (Table 2).

Finally, we noted that 17 (56.7%) of the 30 women had been to other health facilities before admission, whereas the remaining 13 (43.3%) patients sought care at our hospital as their first point of call. Among the 17 women from other facilities, LF was suspected in 8 cases; differential diagnoses included eclampsia, malaria, pyelonephritis, miscarriage, puerperal sepsis, and placenta previa.

## Discussion

LF is endemic to several states in Nigeria, and the number of women at risk for LASV infection during pregnancy is high. Our study highlights the contribution of LF to maternal mortality rates at a teaching hospital in Nigeria, where LF was diagnosed in 44/5,048 (0.87%) of all admitted pregnant women but accounted for 11/84 (13.1%) of maternal deaths at the facility during the study period. The 36.7% LF-related maternal mortality rate we report is roughly in the range of a case series described in Sierra Leone in 1988 (21%) (*11*) and of limited data from Liberia (where the LF-related mortality rate reported before 1973 was 33%–75%) (*24*), although detailed comparison is difficult given the sample sizes involved, decades of separation, and different geographic and genetic contexts.

Most of the deaths in our case series occurred during 2009–2013 (9/19, 47.4%); the mortality rate decreased by more than half for 2014–2018 (2/11, 18.2%). Despite the relatively small numbers in our study, a few key changes occurred in Nigeria, and specifically at ISTH, that are consistent with dropping mortality rates. First, testing of LF and other viral hemorrhagic fevers increased after the 2013–2016 Ebola virus disease outbreak. Second, ISTH has dramatically increased resources devoted to LF research and clinical care. Third, public health activities at ISTH and throughout Edo State increased, including a series of community campaigns and seminars for healthcare workers, with the goal of raising awareness of LF and improving management strategies.

Ongoing public health efforts presume the importance of timely referrals and quick diagnosis of LF, given that poor health-seeking behaviors resulting from lack of awareness are believed to delay medical care and worsen outcomes (*17*,*25*). Furthermore, in our study, 64% of maternal deaths occurred within 24 hours of admission and 100% within 48 hours of admission, suggesting that the disease was often advanced before referrals were made, a finding consistent with previously described delays in care for other obstetric complications (*26*). This observation further emphasizes the need for continuous community engagement, healthcare worker sensitization, an increased index of suspicion in LF-endemic areas, and ready availability of rapid diagnostic tools. The actual contribution of LF to maternal mortality rates is likely underreported in Nigeria, and we suspect LF constitutes a hidden cause of maternal deaths in several LF-endemic communities in a country that already has one of the highest maternal mortality rates in the world (*27*,*28*).

Extravaginal bleeding, convulsions, and oliguria have been found to be associated with poor outcomes in nonpregnant adults (*16*), and we have found similar associations in maternal and fetal prognosis. In addition, we report the concerning finding that more than half of mothers who were admitted to our hospital with a deceased fetus eventually died themselves. This finding is consistent with the experience reported from Sierra Leone in 1988 (*11*), although the causal relationship between intrauterine death and severe LF disease is unknown. The number of cases in our retrospective series was insufficient to compare strategies of evacuation alone, evacuation and ribavirin, or ribavirin alone; further studies are needed to determine the best approach to obstetric care.

Although in Sierra Leone a major correlate of poor outcome was the diagnosis of LF in the third trimester (*11*), we did not observe the same correlation. Many factors might have contributed to this difference; however, we note that the Sierra Leone study endorsed active uterine evacuation in the third trimester and deferred ribavirin (*11*), whereas ISTH uses ribavirin as early therapy in all cases. Because ribavirin has been reported to be teratogenic in animal studies and data on its safety in pregnant women are insufficient (*19*), our decision to use this treatment was not taken lightly. However, although high-quality efficacy trials are lacking and safety data are pending, ribavirin is one of few therapeutic modalities available to us in this situation. The results in terms of maternal and fetal outcomes have been positive in our experience.

The favorable maternal outcome associated with admission with a live fetus suggests the importance of early detection, and we believe the results acquired through our conservative management of these patients challenge the view that active uterine evacuation is required, at least in our setting. Although we did find a significant risk of fetal death overall, we are encouraged because 12 of the 13 women admitted with live fetuses survived the illness, and 11 live births resulted. Our single experience with late uterine death of unexplained cause did increase our surveillance of pregnant survivors of LF, and we now admit such women for more frequent fetal monitoring (biometric and biophysical surveillance) and the option to induce labor in case of fetal compromise.

The generalizability of our findings is limited by a relatively small sample size at a single tertiary-care hospital and the exclusion of several cases because of incomplete records. We further recognize that we lack clinical laboratory data, quantitative RT-PCR assays needed to assess viral load, and pathologic confirmation of cause of death. Although availability of these data points has improved over time at our hospital, we note that they are not always routinely available in our resource-limited setting. We have instead highlighted key clinical findings associated with poor prognosis, including extravaginal bleeding, convulsions, oliguria, and fetal death, that do not require infrastructure for laboratory or pathologic confirmation. In our hospital, women with any of these signs or symptoms receive emergent and aggressive supportive care, including ribavirin therapy.

In conclusion, LF in pregnancy has a high case-fatality rate and is an important and likely underreported cause of maternal death in LF-endemic areas of Nigeria and in other West African countries. Admission to care with a live fetus is predictive of improved maternal outcome, and conservative obstetric management with early ribavirin (instead of evacuation) is producing good outcomes in our hands. Further studies are needed to confirm these conclusions and to provide an updated, data-driven algorithm for clinical management of pregnant women with LF.
